# The Multiplanetary Future of Plant Synthetic Biology

**DOI:** 10.3390/genes9070348

**Published:** 2018-07-10

**Authors:** Briardo Llorente, Thomas C. Williams, Hugh D. Goold

**Affiliations:** 1 Department of Molecular Sciences, Macquarie University, Sydney NSW 2109, Australia; tom.williams@mq.edu.au (T.C.W.); hugh.goold@mq.edu.au (H.D.G.); 2 CSIRO Synthetic Biology Future Science Platform, Canberra ACT 2601, Australia; 3 New South Wales Department of Primary Industries, Orange NSW 2800, Australia

**Keywords:** Synthetic biology, multiplanetary life, habitability of extraterrestrial environments, plants, Mars

## Abstract

The interest in human space journeys to distant planets and moons has been re-ignited in recent times and there are ongoing plans for sending the first manned missions to Mars in the near future. In addition to generating oxygen, fixing carbon, and recycling waste and water, plants could play a critical role in producing food and biomass feedstock for the microbial manufacture of materials, chemicals, and medicines in long-term interplanetary outposts. However, because life on Earth evolved under the conditions of the terrestrial biosphere, plants will not perform optimally in different planetary habitats. The construction or transportation of plant growth facilities and the availability of resources, such as sunlight and liquid water, may also be limiting factors, and would thus impose additional challenges to efficient farming in an extraterrestrial destination. Using the framework of the forthcoming human missions to Mars, here we discuss a series of bioengineering endeavors that will enable us to take full advantage of plants in the context of a Martian greenhouse. We also propose a roadmap for research on adapting life to Mars and outline our opinion that synthetic biology efforts towards this goal will contribute to solving some of the main agricultural and industrial challenges here on Earth.

## 1. Taking Full Advantage of Plants on Extraterrestrial Human Outposts

The exploration of space is one of the most inspiring areas of scientific research and a major driver of technological innovation. Achieving sustainable human presence on alien planetary bodies will expand our understanding of the cosmos, our capacity to investigate fundamental questions, such as the potential for life beyond our home planet, and will enable continued growth of the global economy. Space agencies such as NASA (National Aeronautics and Space Administration) and ESA (European Space Agency) as well as companies from the private sector like SpaceX share the common interest of moving forward the human exploration of deep space and launching the first manned missions to Mars in the near future [1,2,3]. A major factor limiting the expansion of human space exploration is the enormous logistical costs of launching and resupplying resources from Earth. Therefore, developing robust technologies to enable sustainable long-duration human operations in space will be of paramount importance in the coming years.

Human and plant life is intimately linked on planet Earth and so might be true on future extraterrestrial outposts. By supplying oxygen (O_2_), fixing carbon dioxide (CO_2_), and recycling waste and water (H_2_O), plants could contribute to sustaining bioregenerative life support systems [4,5], whilst also providing food and precursors for manufacturing medicines and materials on distant locations such as the Moon or Mars. Making the most of plants on-site would increase self-sufficiency during long-stay residence periods, hence minimizing risks and reducing the transportation of cargo and the deployment of resupply missions. However, because plants have evolved under the conditions of the terrestrial biosphere, substantial resources would need to be allocated if we aim to achieve efficient farming by mimicking Earth’s conditions in other planetary environments. Simple inputs like sunlight and liquid water could potentially be limited, beneficial microorganisms and nutrients might need to be implanted, and well-conditioned greenhouses capable of shielding plants from harmful ultraviolet (UV) and cosmic radiation would need to be built or transported. Considerable energy would also need to be allocated for maintaining additional controlled greenhouse conditions such as temperature, humidity, and pressure. In this context, despite continuous advances in space agriculture [6,7], bioengineering approaches aimed at reducing the burden of sustaining extraterrestrial greenhouses and improving plant performance under different planetary environments remain to be explored.

In this article, we extend the existing body of work on the use of microbes [8,9,10,11] and argue that synthetic biology will provide the means for outpacing terrestrial evolution to take full advantage of plants beyond Earth. We use the forthcoming human missions to Mars as a scenario to discuss a series of bioengineering undertakings that will enable plants to thrive in Martian growth facilities.

## 2. Refactoring Plants for Enhanced Performance on Mars

**Figure 1:**
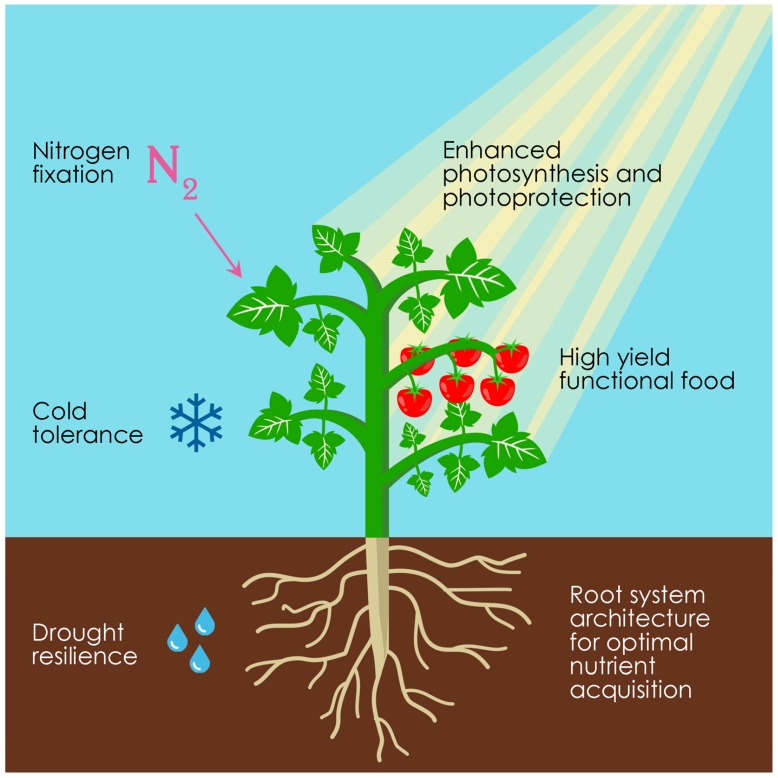
Synthetic biology applied for enhancing plant performance. Different traits that can be engineered simultaneously to take full advantage of plants on Mars (and Earth).

Mars is the most Earth-like of our neighboring planets and the next step for human planetary exploration. It is anticipated that in order to achieve long-duration habitation of the Martian surface, missions to the red planet will need to depart from complete reliance on shipped cargo and achieve a high level of self-sufficiency [8,11,12,13]. As described above, one way to move towards this goal would be to deploy special facilities designed to allow plants to survive the harsh environment of Mars [14,15]. A complementary approach would be to engineer plants for enhanced performance under Martian conditions, an endeavor that will require substantial modifications at multiple levels but will ultimately bring benefits in energy, water, and habitat-space use. In this section, we focus on potential plant synthetic biology solutions to a series of Martian challenges (Figure 1). Because recent studies indicate that the reduced gravity level on Mars of 0.38 g (compared to 1 g on Earth) may not be a major problem for plant growth and development [16], we will not discuss gravity in this article. For a description of the Martian environmental conditions, such as temperature and atmospheric and soil (i.e., regolith) composition, we refer the reader to two recent articles [8,9].

### 2.1. Enhancing Photosynthesis and Photoprotection

Light energy is essential for the photosynthesis process that allows plants to produce oxygen and new biomass from carbon dioxide and water. Plant light energy conversion efficiency is far from being optimal because photosynthetic organisms in the wild have been evolutionarily selected for reproductive success and not for high biomass production [17]. On Mars, where sunlight intensity is significantly lower than on Earth (~43% at comparable latitude and time of day [9]), and where the need of growing plants inside greenhouses will further reduce sunlight levels even if built with the best transparent materials, improved photosynthesis will likely constitute a major advance. Maximizing the use of natural sunlight would save considerable power resources that would have to be otherwise diverted to support artificial lighting [14]. Improving photosynthetic efficiency will, therefore, not only increase plant biomass production but will also translate into energy savings (Figure 1).

Plants harvest energy from a small proportion of the light spectrum, mainly in the wavelength range of 400–700 nm, and thus access only about 50% of the incident solar energy [17]. One promising target for improving plant use efficiency of sunlight would be to expand the region of the light spectrum used by photosynthesis via reengineering the light-harvesting antenna and reaction center complexes [17,18,19,20]. Expanding the spectral coverage of light harvesting towards the UV and/or the infrared regions will mean that photons from currently inaccessible wavelengths would be available for energizing plant growth. Since the absence of a significant ozone layer and low atmospheric pressure of Mars result in a higher surface flux of UV radiation [21], enabling the photosynthetic use of the UV region of the solar spectrum could prove particularly effective. Such a strategy, however, would require the use of UV-transparent greenhouses and also the realization of superior UV-protection mechanisms to minimize cell damage, conceivably by engineering highly efficient synthetic UV-dependent responses. Additionally, since UV is potentially damaging to DNA, RNA, proteins, and cellular metabolism, enhancing UV-tolerance would likely result in a better plant performance in general [22]. Along the same lines, as photooxidative stress occurs when the absorbed light energy exceeds that used in photosynthesis, engineering improved photoprotection mechanisms could further enhance the performance of the light-harvesting machinery [23], as recently demonstrated in tobacco (*Nicotiana tabacum*) plants [24].

One other ambitious approach for improving plant biomass production involves increasing the yield of photosynthetic carbon assimilation. There are numerous strategies that are currently being pursued towards this goal, from improving the catalytic activity of Rubisco (i.e., ribulose-1, 5-bisphosphate carboxylase/oxygenase, the CO_2_-fixing enzyme in photosynthesis) and implementing CO_2_-concentrating mechanisms to engineering photorespiration bypasses and installing synthetic carbon fixation pathways [20,25,26,27,28]. Among these strategies, building new-to-nature CO_2_-fixing pathways holds the most promise to improve photosynthetic light energy conversion, since it is arguably the approach that would be less likely limited by serendipitous evolutionary constraints [29,30]. Also, given that CO_2_ and O_2_ compete at the active site of Rubisco and that the atmospheric CO_2_/O_2_ ratio on Mars is enormously higher than on Earth [8], it is possible that carbon fixation by Rubisco on Mars could be highly effective.

### 2.2. Improving Drought and Cold Tolerance

Water will be a crucial resource for plants but also for many other applications in a Martian outpost [31]. Because water on Mars is mostly available in the form of ice [32], part of the energy budget of the outpost will need to be allocated for its extraction and recycling [31]. Also, if energetically expensive systems (e.g., hydroponics) were required to sustain plant growth, additional energy will need to be diverted for this purpose [6,15,33]. Developing plants that require less water per unit mass of production will, therefore, contribute to better water and energy management on Mars (Figure 1).

One way to improve drought tolerance would be to manipulate the opening and closure of stomatal pores, from which water is lost via transpiration. This approach has already been shown to reduce plant water loss [34] and could potentially be even more effective if the signaling pathways that adjust stomatal behavior in response to drought were rewired to achieve programmable functional insulation, hence minimizing growth penalties often derived from crosstalk between stress responses and developmental networks [35,36,37]. Other promising approaches would be to engineer plants of interest with crassulacean acid metabolism, which increases water-use efficiency and enables plants to inhabit water-limited environments such as semi-arid deserts [38]. More progressive synthetic biology approaches could even enable the engineering of drought-tolerance mechanisms analogous to those found in resurrection plants, extremophytes that can withstand severe drought conditions [39,40], or even more evolutionary distant organisms that can undergo anhydrobiosis and survive extreme desiccation [41,42].

Mitigating the low average temperature of Mars and its huge diurnal thermal variation [43] will be another key aspect that will require substantial energy allocation [14]. Engineering cold-hardy plants could help reduce the amount of energy allotted to meet the thermal requirements of Martian greenhouses (Figure 1). As with the case of improving drought tolerance, engineering plants of interest to exploit the protection mechanisms used by other organisms adapted to withstand low temperatures is a promising strategy. There are clear examples that cold tolerance can be enhanced by the expression of ice-binding proteins capable of inhibiting the growth of damaging ice crystals [44]. Increased levels of membrane unsaturated fatty acids and certain osmoprotectants (e.g., fructans) also lead to cold tolerance; hence manipulating their metabolism is also a particularly attractive target [45,46]. A more sophisticated strategy would be to design a dynamic multilevel cryoprotective response regulated, perhaps through the circadian clock [47], in such a way to anticipate the large temperature drop of the Martian night. Synthetic circadian regulation could enable optimal use of energy by timing the cryoprotective response with the diurnal solar oscillation [48]. Because the length of Mars day (~24.5 h) is similar to that of Earth (~24 h) [3] just minor adjustments of the plant circadian timing system might be required for optimal functioning.

### 2.3. Engineering High Yield and Functional Food

The limited size of the Martian greenhouses and the availability of indispensable plant nutrients like phosphorus and nitrogen will represent additional challenges for an agricultural system on Mars. Ideal plants should have high biomass productivity, high harvest indices, minimum horticultural requirements, and provide food for a functional diet [13,49] (Figure 1).

A possible solution to boost biomass productivity would be to achieve cultivation at very high plant density [50,51] by manipulation of the shade avoidance response [52], which can be detrimental to yield because carbon resources are redirected to stem or petiole elongation at the expense of biomass production [53]. Redesigning the plant development and architecture at different levels could also lead to crop variants with extraordinary harvest indices. This idea has recently been demonstrated in tomato (*Solanum lycopersicum*) plants engineered in a number of architectural traits resulting in improved productivity [54,55,56]. Moreover, engineering the root system architecture for optimal nutrient acquisition and increased fertilizer use efficiency could further translate to higher yields [57,58] (Figure 1). Changing the root system architecture to improve phosphorus uptake [59] could be particularly relevant on Mars, as it is unclear if phosphorus is readily available to sustain plant growth [8,60,61], in which case, it should be supplemented as a fertilizer.

Nitrogen, which is also an essential plant nutrient, is present on the surface of Mars in the form of nitrate [62] that could potentially be biochemically accessible to plants. Alternatively, nitrogen gas (N_2_) could be directly assimilated from the Martian atmosphere [8]. However, because the capacity to fix gaseous N_2_ is restricted to a specialized group of prokaryotes and does not occur in plants, one ambitious goal would be to endow plants with the capacity to directly assimilate atmospheric nitrogen (Figure 1). To this end, all the required microbial machinery for fixing nitrogen could be transferred into plants, a strategy that is currently being pursued by different laboratories and, although technically challenging, it is certainly within the capacity of modern synthetic biology [63,64,65,66,67,68]. However, given the huge difference in the atmospheric nitrogen content of Earth (~78%) and Mars (~2.7%) [9], such a transplanted microbial pathway might not work efficiently on Mars. Possible solutions to this challenge would be to exploit indigenous Martian nitrogen [62] to enrich greenhouse N_2_ concentration or to employ protein-engineering techniques to increase nitrogenase affinity and develop a N_2_-fixation pathway of high performance under low nitrogen concentration. Alternatives to endowing plants with the capacity to assimilate atmospheric nitrogen would be to engineer nitrogen fixation in root-associated microbes or to develop synthetic root-microbe symbiosis with microorganisms already capable of fixing nitrogen [69,70,71].

Another consideration for sustaining an extended human presence on Mars is that of producing nutritious food. Poor nutrition can cause detrimental effects on health and adversely affect physical and cognitive performance [72]. Plants could be central to maintaining good nutrition on long-duration manned space expeditions. For instance, the consumption of carotenoids, a group of isoprenoid compounds with activity as antioxidants and vitamin A precursors [73], has been identified as of particular interest for humans on space [49,74]. Unlike plants, which synthesize carotenoids in their plastids [75,76], humans do not produce carotenoids and have to incorporate them in their diets [73]. Because carotenoid accumulation in plants is the result of multiple processes [23,76,77], the combination of various bioengineering strategies, from manipulating the carotenoid biosynthesis and storage mechanisms to installing alternative carotenogenic pathways [76,78,79,80,81] holds potential to take the carotenoid content of crops to a new level. From a holistic point of view, the ultimate synthetic biology approach to make the most of plant-based food on Mars would be to develop multi-biofortified crops with improved nutritional properties [82,83,84] and enhanced quality traits (e.g., extended shelf life and reduced allergenicity) [85,86,87,88,89,90,91,92].

## 3. Tailoring Microorganisms to Complement and Facilitate Plant Life on Mars

**Figure 2:**
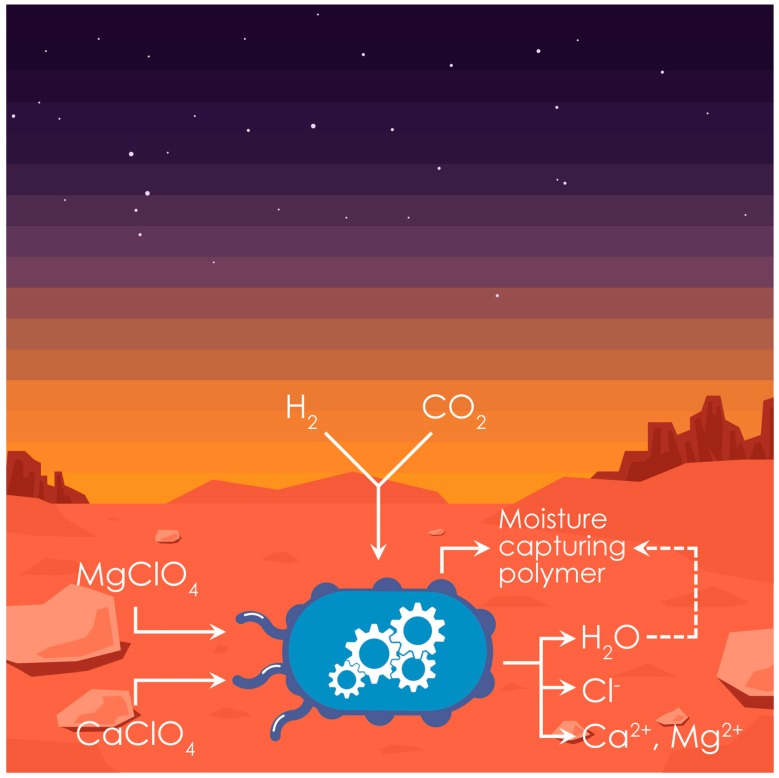
Engineering microorganisms to facilitate plant life on Mars. This conceptual microbe scavenges atmospheric hydrogen (H_2_) and carbon dioxide (CO_2_), and it is customized to condition Martian soil for plant growth by reducing soil perchlorate salts (MgClO_4_ and CaClO_4_) and increasing soil moisture. H_2_O: water; Cl^−^: chlorine; Ca^2+^: calcium; and Mg^2+^: magnesium.

The establishment and utilization of plants on Mars would benefit significantly from its use in conjunction with microorganisms. Besides their potential use to supply nitrogen as discussed above, engineered microbes would be necessary for the removal of toxic compounds from the Martian soil and its transformation from an arid and oligotrophic desert material into a nutrient-rich soil able to support plant growth (Figure 2). As in the case of plants, due to the drastically different environment in which these microbes would need to perform, synthetic biology will be essential for engineering desired functions. Once plants are established, microbes could be used to convert plant biomass into proteins and metabolites that serve as materials, chemicals, and medicines. By using plant sugars and biomass as versatile feedstock for bioprocessing, these resources could be made available on-demand at high rates, titers, and yields. It is important to note that an enormous array of microbes designed to perform a multitude of useful tasks could be transported to Mars with very little cargo-burden. In this section, we focus on crucial applications of microorganisms relating to the establishment and utilization of plants on Mars.

### 3.1. Conditioning Martian Soil for Plant Growth Using Microbes

Recent experiments have shown that several plant species are remarkably healthy when grown on Mars soil simulants [93]. Additional experiments simulating the gravitational conditions of Mars also suggest that soil-based agriculture would require about 90% less water than on Earth as a consequence of lower leaching rates [94]. As a whole, these results are encouraging for the prospect of utilizing Martian soil for growing plants on Mars. Towards this goal, microorganisms can be utilized to perform several critical tasks in the conditioning of Martian soil for plant growth.

First, we need to identify and learn from microorganisms capable of surviving Martian soil conditions with minimal nutritional requirements. The Antarctic Dry Valleys on Earth have some of the most comparable conditions to Mars, with extreme aridity, low temperatures, high radiation, and lack of nutrients [95,96]. It was recently found that a novel mode of metabolism facilitates bacterial persistence in these extreme conditions, whereby atmospheric trace levels of hydrogen (H_2_), CO_2_, and carbon monoxide (CO) provide energy and carbon to support microbial communities [97]. This type of metabolism, for example, could potentially be exploited by primary colonizing microbes designed to implement the first conditioning steps of the hyper-arid Marian soil. Critically, the required gases to sustain this type of chemotrophic growth could be made available directly from the Martian atmosphere and water electrolysis [8].

The Martian soil has been found to contain high levels of perchlorates [98]. Perchlorates are toxic to human hormone systems, and any soil used to grow plants for human consumption would need to have dramatically lowered perchlorate levels [99,100]. One way to achieve this would be to remove perchlorate salts with water; however, this would impose a burden on the valuable water and energy resources on Mars. An attractive alternative solution to this problem is to use biological removal of perchlorate by engineering CO_2_-utilizing bacteria to express perchlorate reduction enzymes. This would enable continued bioremediation over time and possibly contribute to bacterial growth. Alternatively, bacteria capable of complete perchlorate reduction [99] could be engineered for autotrophic carbon fixation, although this would be a far more complex feat. In addition to detoxifying Martian soil, the biological reduction of perchlorates would have the additional benefit of releasing water found as hydrated perchlorate salts [101], increasing soil moisture (Figure 2). To further improve soil water content, bacteria could be engineered to produce an extracellular polysaccharide or adhesive protein that would bind soil particles together and hence mitigate desiccation [102] (Figure 2).

### 3.2. Microbes for Metabolite and Protein Production from Plant Material

In recent years, a burgeoning bio-economy has emerged where the precision, reaction rates, and diversity of microbial biochemistry have been harnessed using synthetic biology to produce a multitude of industrial and consumer products. This economy is arising to create products that are either produced unsustainably from oil (e.g., chemicals) [103], inefficiently from plants and animals (e.g., medicines) [104] or that cannot be produced industrially in any other way (e.g., spider silk) [105]. The advantages of this paradigm will be even more pertinent on extraterrestrial outposts, where every resource must be consumed and/or produced as efficiently as possible. Additionally, any biomolecule that can be produced on-site and on-demand lowers the burden of having to be transported. Many of these valuable biomolecules should, if possible, be produced autotrophically using photosynthesis, acetogenesis, or methanogenesis from waste carbon dioxide and carbon monoxide [8,9,10,11,12]. However, these modes of metabolism have limitations in terms of the production rates, titers, and yields of specific products depending on the adenosine triphosphate (ATP) and redox requirements of a given production pathway [106,107,108]. Using aerobic heterotrophic catabolism of plant-derived sugars and biomass can circumvent these limitations due to greater ATP generation and redox flexibility per molecule of substrate. Such a production scenario could, therefore, be advantageous and compatible with growing plants on Mars. Using plant biomass to provide sugars for fermentation would also afford great versatility and adaptability, as the same renewable feedstock would serve as input to a variety of products as they are required. This mode of production would also enable the use of the highly developed synthetic biology and bio-production tools available in model organisms such as *Escherichia coli* and *Saccharomyces cerevisiae*.

Production of medicines on Mars will be particularly important to reduce cargo transport and avoid degradation of stored medicines by radiation and temperature variations [109]. Synthetic biology principles could be applied to efficiently and simultaneously produce many pharmaceutical molecules on-demand using both plant biofactories [110,111] and compact microbial bioreactors [112,113,114]. While plants are very attractive for the production of medicines for oral delivery because fermentation and purification processes can be avoided [115], microorganism such as *Pichia pastoris* are advantageous synthetic biology chassis organisms for a multitude of other applications due to their metabolic versatility and extensive gene-engineering tools [116]. *P. pastoris* could potentially be an ideal production host for medicines, metabolites, and materials on Mars and it also has the ability to grow on methanol as a sole carbon source. Methanol is a one-carbon alcohol that can be derived from the oxidation of methane or the reduction of CO/CO_2_ with H_2_ to give methane, and then methanol. Given that CO/CO_2_ and H_2_ can be obtained from the combustion of organic material such as inedible plant matter or human waste, the Martian atmosphere, and water electrolysis, methanol derived from these sources is a potentially versatile and easily storable carbon source for microbial production strains.

## 4. A Roadmap for Research on Adapting Life to Mars

**Figure 3:**
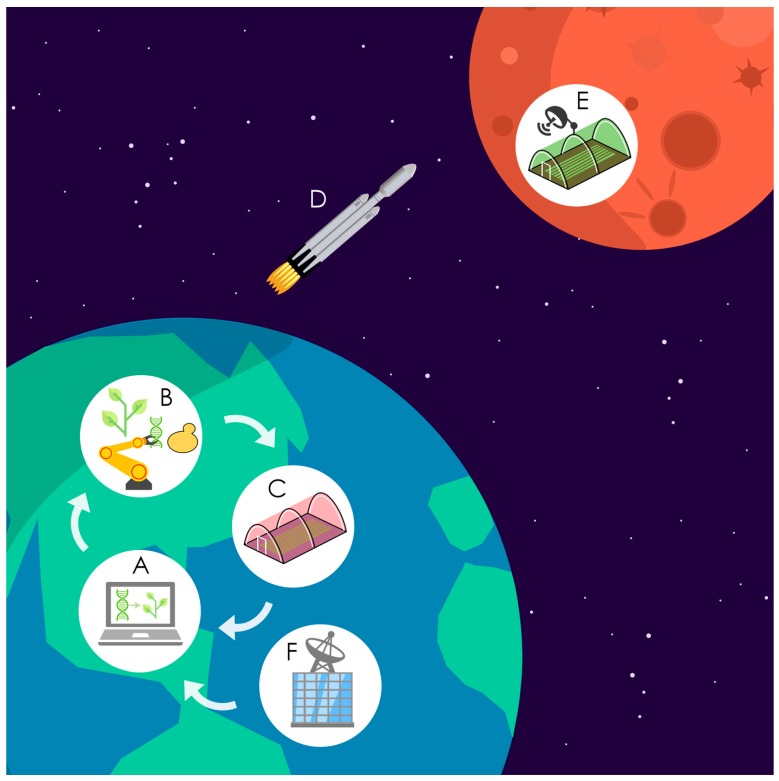
Schematic roadmap for research on adapting life to Mars. The Mars Biofoundry integrates the design of synthetic biology approaches (**A**) with an automated platform for implementing bioengineering designs in plants and microbes (**B**) and a facility for high-throughput phenotyping under simulated Martian conditions (**C**). The process iterates as a design-build-test cycle. Eventually, engineered organisms could be periodically transported to Mars (**D**) to perform experiments within miniature growth facilities (**E**). Remote monitoring of performance on Mars (**F**) would provide critical knowledge to adjust the work carried out at the biofoundry on Earth.

Achieving the proposed goals of adapting plant and microbial life to thrive on a Martian environment in a timeframe compatible with the forthcoming human expeditions to the red planet will require novel approaches. We propose that this formidable challenge can be tackled by establishing a ‘Mars Biofoundry’, that is, an automated and versatile platform capable of expediting the engineering and high throughput phenotyping of biological systems adapted to the environmental conditions that will be encountered on Mars (Figure 3).

Biofoundries facilitate complex automated workflows to build, analyze, and optimize thousands of bioengineering designs in parallel, hence accelerating the exploration of enormous design-space in ways that are unfeasible with traditional approaches [117]. While the majority of current platforms operate with microorganisms, the Mars Biofoundry would also incorporate plants. Therefore, it should be capable of efficient engineering and screening of high-performing plants and microbes under simulated Martian conditions. This unique capacity would also help to identify plant species that would be best suited for Mars.

Direct engineering of plants, even if implementing the most advanced methods [118,119,120], might be impractical because of their lengthy regeneration times and the sheer size of facilities to house large-scale screens. A far more progressive approach would be to test plant-targeted bioengineering designs in heterologous organisms that would be easy to manipulate, capable of rapidly generating large populations, and suitable for massive functional analysis in parallel [64,121,122,123]. Microorganisms such as algae, yeast, and bacteria could be used to rapidly test an enormous array of circuit and pathway designs and, whenever possible, also as plant-proxies in screens simulating the conditions of Mars. Because traits linked to plant development are unlikely to be characterized in unicellular microorganism, the best-performing solutions would then be transferred into simple multicellular plant models such as *Marchantia polymorpha* and *Physcomitrella patens* [124,125] for additional characterization under simulated Martian greenhouse conditions (Figure 3). Further refinements in planta (e.g., traits related to functional or anatomical tissue differentiation) or via reiterative microbial engineering rounds could be implemented if necessary. The whole process of outsourcing the optimization of plant-targeted bioengineering designs to microbes could be completed in far less time and with only a fraction of the cost that would be required if pursuing the direct engineering of plants.

Ultimately, we envision that shakedown experiments could be performed within miniature growth facilities deployed on the surface of Mars every ~2 years by future frequent unmanned flights [3]. Remote monitoring of performance on Mars would provide critical knowledge to adjust the work of the biofoundry on Earth (Figure 3). Research on adapting life to Mars would also help to assess the risk of planetary biological contamination in case of accidental release [9] and would, therefore, be invaluable to design effective strategies aimed at reducing this risk.

## 5. From Earth to Mars and Back to Earth

The human exploration of Mars will be one of the greatest achievements of humanity and the first step of our multiplanetary journey. Developing the technology required for sustaining humans on another planet would lead to revolutionary advancements and fascinating scientific discoveries. Plants could contribute to this enterprise with great implications for Earth. A growing global population is leading to rising demand for food, which will require an increase in agricultural productivity without adverse environmental impact and without placing more land under cultivation [126]. Crop yields are already reaching capacity [127] and continuation with current agricultural technology will strain Earth’s ecosystem [128]. Improving plant traits useful for Mars such as those discussed earlier (Figure 1) will have far-reaching implications across the board for terrestrial agriculture. Advances in microbial-mediated soil conditioning, which will be required for facilitating plant life on Mars (Figure 2), and in the use of plant biomass as renewable feedstock for the manufacture of all kinds of products will respectively help improve crop yield and develop a truly sustainable industry on Earth. Establishing facilities such as the proposed Mars Biofoundry (Figure 3) will likely bring immense benefit to the turnaround time of plant research, hence having widespread implications for addressing the needs of food security and environmental protection but also advancing our understanding of plant biology. Ultimately, the main beneficiary of efforts to develop plants for Mars is Earth.
